# Transplantation of Hepatitis B Surface Antigen Positive or Viremic Kidneys: A Systematic Review and Meta‐Analysis

**DOI:** 10.1111/ctr.70733

**Published:** 2026-11-03

**Authors:** Dhruv Ahuja, Beatrice P. Concepcion, Yuhong Yuan, Rachel Forbes, James L. Rogers, Helen S. Te, Yvonne El Kassis

**Affiliations:** ^1^ Department of Medicine Indira Gandhi Hospital New Delhi India; ^2^ Section of Nephrology, Department of Medicine University of Chicago Medicine Chicago Illinois USA; ^3^ Department of Medicine McMaster University Hamilton Ontario Canada; ^4^ Department of Medicine Western University London Ontario Canada; ^5^ Division of Kidney and Pancreas Transplantation, Department of Surgery Vanderbilt University Medical Center Nashville Tennessee USA; ^6^ Vanderbilt University School of Medicine Nashville Tennessee USA; ^7^ Section of Gastroenterology, Hepatology, and Nutrition University of Chicago Medicine Chicago Illinois USA

**Keywords:** donor disease transmission, hepatitis B, hepatitis B transmission, graft and patient survival, kidney transplantation

## Abstract

**Purpose:**

This systematic review and meta‐analysis examines the evidence related to the use of HBV positive kidneys into non‐viremic kidney transplant recipients (KTRs).

**Methods:**

We searched PubMed and Embase through November 2025 and identified studies of recipients of hepatitis B surface antigen positive (HBsAg+) or viremic kidneys. Studies with a comparison group of KTRs of HBV negative kidneys that reported on patient and allograft survivals were included in the meta‐analysis. The risk ratios (RR) for patient and allograft survivals were pooled to obtain the estimate and 95% confidence interval, at 1‐, 3‐, and 5‐years post‐transplant. Viral transmission including HBsAg development, HBV viremia, or hepatitis was also reviewed.

**Results:**

Eleven studies were included in the systematic review and 6 in the meta‐analysis. They included 638 KTRs of HBV positive kidneys and 2800 recipients of HBsAg negative non viremic kidneys. At 1, 3, and 5 years, the RR of allograft survival was not statistically different between the 2 groups and was 1 [0.98–1.01], 1.01 [0.98–1.04], and 1.00 [0.91–1.09] respectively. Similarly, the RR of patient survival was comparable between the 2 groups. The rate of HBV transmission was low (4.94%), especially when prophylaxis was used and when recipients were immune pre transplant.

**Conclusion:**

Kidneys from HBV infected donors confer similar patient and allograft survival than those from HBV negative donors. They may be used to expand the donor pool, in immune KTRs and with appropriate prophylaxis, in light of this emerging evidence of safety with very low reported rates of transmission.

**Abbreviations**
Anti‐HBc: hepatitis B core antibody
CI: confidence intervals
HBsAb: hepatitis B surface antibody
HBsAg: hepatitis B surface antigen
HBIG: hepatitis B immunoglobulins
HBV: hepatitis B virus
OPO: organ procurement organization
OPTN: Organ Procurement Transplant Network
NAT: Nucleic acid amplification testing
RR: risk ratio
UNOS: United Network for Organ Sharing

## 1 Introduction

The striking imbalance between the number of patients waiting for a kidney transplant and the number of available deceased donor (DD) kidneys has led to the development of strategies to increase the DD pool and address the organ shortage. These include the use of kidneys with high kidney donor profile index (KDPI) or acute kidney injury, and the more novel use of kidneys from donors with infections where transmission can be prevented or easily managed in the recipient post‐transplant. For instance, the THINKER trial reported the safe use of kidneys from Hepatitis C (HCV) viremic donors into non viremic recipients, which was made possible due to the availability of direct acting antivirals that result in a sustained virologic response of 97.7% when taken over the course of 12 weeks [1].

Kidneys from donors infected with hepatitis B represent another potential opportunity to expand the donor pool, especially that one‐third of the global population has serological evidence of past or current infection with hepatitis B [2]. Hepatitis B virus (HBV) is a double‐stranded enveloped DNA virus of the family of *Hepadnaviridae* that replicates mostly in the liver, although extrahepatic replication may also occur and the virus can also be detected in the blood. It is transmitted parenterally through percutaneous or mucosal exposure to infected blood or other body fluids. In adults, acute HBV infection can result in asymptomatic infection, self‐limited hepatitis (a third of acute infections are symptomatic), and very rarely, fulminant hepatitis (< 1%). Resolution of HBV with hepatitis B surface antigen (HBsAg) clearance or seroconversion occurs in more than 95% of adult patients and only 5% of patients progress to chronic hepatitis B, defined by the persistence of HBsAg for more than 6 months. Chronic hepatitis B is a risk factor for cirrhosis and hepatocellular carcinoma, and the risk increases proportionally with increasing viral loads. There are currently 850,000 people in the United States (US) who are living with chronic hepatitis B. The actual number is estimated to be between 730,000 to 2.2 million, with an estimated prevalence of 0.3 to 0.7% [3].

The Organ Procurement Transplant Network (OPTN) policy issued in 2020 requires that all DDs be tested for HBsAg, anti‐HBc, and HBV DNA within 96 h of donation. In 2015, The American and Canadian Society of Transplantation published consensus guidelines for management of organ recipients following the use of organs from HBV positive donors [4]. While they recommend considering organs from anti‐HBc+ donors for all adult transplant candidates, they recognize that very few organ procurement organizations (OPOs) and transplant centers in the US use organs from HBsAg+ donors. However, the more recent development of highly efficacious vaccines against hepatitis B, coupled with potent antiviral therapies, has made the use of kidneys from HBV viremic or HBsAg positive donors appealing for some transplant centers in the US and China [5, 6, 7, 8, 9].

The purpose of this systematic review and meta‐analysis is to evaluate the currently available evidence related to the transplantation of kidneys from donors with hepatitis B viremia or HBsAg positivity into non‐HBV viremic kidney transplant recipients (KTRs), to define best practices that would allow for a safe and successful real‐world use of such organs, and to identify potential gaps in this practice.

## 2 Methods

This systematic review and meta‐analysis was conducted in accordance with the Preferred Reporting Items for Systematic Reviews and meta‐analysis (PRISMA) 2020 checklist [10]. We included studies of KTRs who received a kidney transplant from a donor who was HBsAg positive or was viremic [HBV nucleic acid (NAT) positive]. The primary outcome of interest was allograft and patient survival at 1, 3, and 5 years. The secondary outcome of interest was HBV transmission, a surrogate marker of the safety of this practice. However, due to inconsistencies in reporting across the various studies, viral transmission was reviewed and included in the systematic review but not in the meta‐analysis. Due to the differences in hepatitis B genotypes prevalence between China and the US, with subsequent differences in natural history of the disease and risk of liver cirrhosis and HCC, we performed additional subgroup analyses to separate the studies from the US and those from China.

### 2.1 Literature Search and Selection Criteria

We performed a systematic literature search across multiple electronic databases including Pubmed and Embase, from inception to November 2025. We utilized a combination of keywords and controlled vocabulary of search (MeSH or Emtree terms) to identify studies with reported data on HBsAg or NAT status of the donor kidney at the time of transplantation and post‐transplant outcomes.

For the meta‐analysis, we included studies that met the following inclusion criteria: *Population‐* adult patients with chronic kidney failure, with or without dialysis, who received a kidney transplant; *Exposure‐* KTRs receiving a kidney from a living or DD who is HBsAg or NAT positive; *Comparator‐* KTRs receiving kidneys from HBsAg and NAT negative donors; *Outcome‐* graft and patient survival at 1, 3, and 5 years. We excluded the following: 1) non‐comparative studies; 2) studies with <1 year follow‐up period; 3) review articles and guidelines; 4) studies analyzing patients from the same database and having an overlapping period of assessment from that database; 5) conference proceedings or unpublished studies.

We identified additional studies which did not have a comparator group, or which did not comment on patient and allograft survival at 1,3, and 5 years and we included them in the systematic review but not in the meta‐analysis.

Two authors [DA and YK] independently screened the titles and abstracts of the studies then reviewed the full text of those meeting the eligibility criteria. Any disagreement was resolved by an independent investigator [BPC]. Data extraction was performed independently by 2 authors on a pre‐specified data abstraction form. The following data were abstracted: 1) study characteristics: primary author, year of publication, study period, country where the study was conducted, study design; 2) recipient and donor characteristics including type of donor transplant (living vs DD), recipient and donor viral serologies which included HBsAg and hepatitis B surface antibody (HBsAb) status; 3) peri‐transplant prophylaxis with hepatitis B immunoglobulins (HBIG) or antivirals; 4) primary outcomes: number of grafts and number of patients survival at 1, 3 and 5 years; 5) secondary outcomes: number of patients with viral transmission including HBsAg development, HBV viremia, or hepatitis.

### 2.2 Statistical Analysis

We utilized random effects meta‐analysis approach described by Hartung‐Knapp and restricted likelihood meta‐analysis to obtain proportions and 95% confidence intervals (CI) [11]. We calculated the pooled risk ratios (RR) and 95% CI for each outcome. The estimates and standard error from individual studies were assessed using inverse variance method before pooling. The Hartung‐Knapp method adjustment was preferred as it provides more conservative and accurate CIs. Heterogeneity across studies was assessed using I^2^ statistic, which quantifies the percentage of total variation across studies attributed by heterogeneity across studies. I^2^ value of < 30%, 30%–60%, 60%–75% and > 75% were suggestive of low, moderate, substantial and considerable heterogeneity, respectively. Subgroup analyses based on the geographical location of the study were performed. Publication bias was assessed through funnel's plot and egger's test, but those were not included as there were less than 10 studies in the meta‐analysis [12]. Risk‐of‐bias assessment was evaluated by the Newcastle‐Ottawa Scale (NOS) (supplemental material). All analyses were conducted using R 4.3.2. using package ‘meta’.

## 3 Results

The initial search yielded 1337 studies, which mostly included duplicates, and ultimately resulted in 70 studies that were screened by title and abstract and 42 studies that were reviewed in detail. Of these, 11 were included in the systematic review. Six of the 11 studies reported graf survival beyond one year and were included in the meta‐analysis. Figure 1 illustrates the study selection flowsheet. Table 1 summarizes the studies included in the meta‐analysis.

**FIGURE 1:**
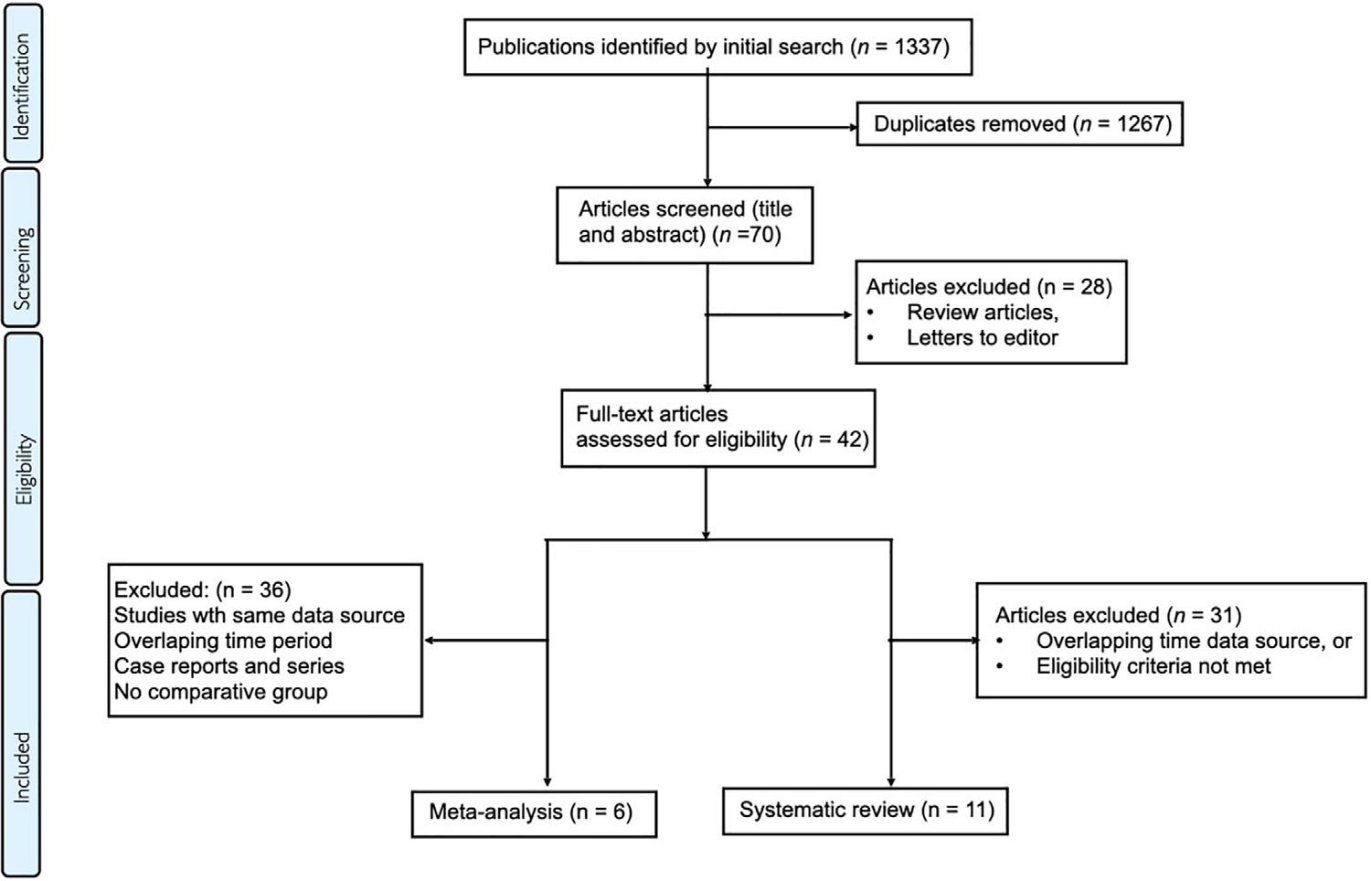
PRISMA.

**TABLE 1:** Summary table of the 11 studies of recipients of HBV positive kidneys that were included in the systematic review.

Study Year PMID Country	N of recipients of HBsAg or NAT + kidneys	Design (Txp years)	Mean follow up period	Donor type	Percentage of viremic donors	Recipient HBsAb positive (%)	Prophylaxis (%)	Hepatic events (%)	Allograft survival at 1 year (%)	Patient survival at 1 year (%)
Lopez‐Soler 2024 38761060 USA	16	Prospective (2020–2023)	Not specified. Only 8/16 recipients reached the 1‐year mark	DD	100	100	Entecavir for 1 year in all recipients	3 (18.7%) patients had transient HBV viremia at 1 week but viral load was undetectable at 9 and 12 months	N/A	N/A
Singhal 2023 **37953470** USA	310	Retrospective (1999–2021)	3.7 year	DD	N/A	67.9	N/A	N/A	90.8	94.4
Yin 2023 **37592870** China	105	Retrospective (2017–2020)	25 (1–46) months	LD 57.1%, DD 42.9%	79	76.2	HBIG alone: 34.2% Antiviral alone: 29.5%, HBIG and antiviral: 36.2%	DNH: 3.8, Denovo anti‐HBc: 58	100	98.99
Carnemolla 2022 **35642883** USA	8	Retrospective (2017–2020)	11.2 months	DD	100	100 % had titers > 10 mIU/mL	88% received antivirals (Entecavir or Lamivudine)	62.5 % had anti‐HBc seroconversion at an average of 47 days post txp	N/A	N/A
Wang 2021 **32100025** China	83	Retrospective (2009–2017)	36 (6–106) months	LD	34	75.9	HBIG: 51% Antiviral: Lamivudine or Entecavir for 1–3 months post txp: 78%	HBsAg development: 2.4. They were nonimmune at the time of transplant	98.8	97.6
Delman 2021 **34506310** USA	56	Prospective (2017–2020)	1 year	DD	100	60.7	Entecavir for 1 year for all recipients	Transient HBV viremia, without hepatitis, which resolved after a median of 80 days of Entecavir: 16.4	89.5	94.4
Yilmaz 2015 **26156252** Turkey	26	Retrospective (2006–2014)	N/A	LD 92.3%, DD 7.7%	100	N/A	Lamivudine for 6–12 months for all recipients	Acute hepatitis infection: 11.5. They were immune.	95	100
Chancharoenthana 2014 **25395260** Thailand	43	Retrospective (2000–2013)	58.2 (16.7–158.3) months	DD	0	100% had titers > 100 mIU/mL	Antiviral (Lamivudine): 48% Lamivudine + HBIG: 4.6%, None: 46.5%	None (no HBsAg, no anti‐HBc conversion, no HBV viremia)	N/A	N/A
Tuncer 2012 22841231 Turkey	35	Retrospective (2008–2011)	N/A	LD	0	100 % had titers > 10 mIU/mL	None	None	N/A	N/A
Jiang 2009 **19538486** China	65	Prospective (2002–2006)	30.2 ± 11 months	DD	10.7	100	HBIG 400 units monthly x 2 (100%), 7 recipients of HBV DNA + kidneys received HBIG weekly for 3 months and Lamivudine for 6 months	1 patient (1.5%) developed HBsAg with transaminitis but remained HBV DNA negative, 7 recipients developed anti‐HBc	98.5	100
Berber 2005 16387070 Turkey	7	Retrospective (over 11 years)	30 (16–121) months	LD 57.1%, DD 42.9%	0% in the LDs, Unknown in the DD	100	Lamivudine for 1–3 years, only in recipients of DD kidneys because their NAT status was unknown	None	100	100

Abbreviations: DD, deceased donor; DNH, de novo hepatitis; HCC, hepatocellular carcinoma; LD, living donor; Txp, transplant.

Hepatic events included hepatitis, HBV viremia, HBsAg development, and HCC. None of the patients had fulminant hepatitis, liver failure, HCC, or cirrhosis.

### 3.1 Systematic Review

Table 1 summarizes the characteristics of the studies included in the systematic review. There were 11 studies with a total of 754 recipients of HBV positive kidneys (HBsAg+ and/or viremic kidneys) between the years of 1994 and 2023 [5, 6, 7, 8, 9, 13, 14, 15, 16, 17, 18]. All studies were observational. The majority were retrospective in nature and only 3 were prospective [5, 9, 14]. Majority included patients in the USA, China, Thailand, and Turkey. Mean follow up time was 30 months (range 1‐158 months).

Of the 754 recipients of HBV positive kidneys, 549 (72.8%) received a DD kidney and 205 (27.2%) received a living donor kidney. While most of the studies included recipients who were immune to HBV with documented anti‐HBs titers pre‐transplant, almost a quarter (22.9%) of the recipients of HBV positive kidneys were not immune at the time of the transplant. The studies reported on the prophylactic strategies, viral transmission, and hepatic events except for a retrospective cohort study of the United Network for Organ Sharing (UNOS) database which lacked this level of granularity. In addition, a study from Turkey of 35 immune recipients of living donor HBsAg positive but non‐viremic kidneys did not include information on recipient prophylaxis. Amongst the remaining 9 studies commenting on the prophylactic strategies in 409 recipients of HBV positive kidneys, 185 patients (45.2%) received perioperative HBIG and 271 patients (66.2%) were started on antivirals (lamivudine or entecavir). The prophylactic strategies were not standardized and the number of doses of HBIG and the duration of antiviral therapy post‐transplant were variable within a given study, ranging from 1 month to 3 years post‐transplant. Information on viral transmission and hepatic events was provided in 10 studies which included a total of 444 patients. Twenty two recipients (4.9%) of HBV positive kidneys had evidence of viral transmission including transient HBV viremia, HBsAg development, transaminitis, or clinical hepatitis. However, the largest study which included 310 KTRs of HBV positive kidneys did not comment on the transmission rate and only commented on the patient and allograft survival, and the above reported transmission rate of 4.9% was derived from the remaining 444 recipients of HBV positive kidneys included in the systematic review. Importantly, reported viremia events were transient in nature in the vast majority of the cases, and most studies reported complete resolution of the viremia at the time of last follow up. Very few studies commented on the development of denovo HBc antibodies. In one study that did, 58% of the recipients of HBV positive kidneys developed anti‐HBc, implying an immunological response to HBV exposure.

### 3.2 Meta‐Analysis

There were 6 studies that commented on patient and allograft survivals post‐transplant and those were included in the meta‐analysis. All studies were observational, four were retrospective and two were prospective. There were a total of 638 KTRs who received a HBsAg positive or viremic kidney and were compared to a control group of 2800 recipients of HBsAg and NAT negative kidneys. The mean recipient age in the HBV positive kidney group was 42.8 years and 29.9% were female, whereas it was 46.6 years and 48.29% were female in the control group.

#### 3.2.1 Graft Survival Rates

At 1‐, 3‐, and 5‐years post‐transplant, the relative risks (RR) of allograft survival were not statistically different between the two groups across all the studies. The overall graft survival rates were 94.2%, 89.2%, and 78.2% at 1‐, 3‐, and 5‐ years in the group of patients receiving HBsAg or NAT positive kidneys compared to 95.6%, 88.6%, and 76.5% in the control group (Figure 2). The RR of allograft survival in the recipients of HBsAg or NAT positive kidneys compared to the control group was 1 [0.98–1.01], 1.01 [0.98–1.04], and 1 [0.91–1.09] at 1‐, 3‐, and 5‐years post‐transplant, respectively (Figure 3). The graft survival showed low heterogeneity across all time points, I^2^ of 0% at 1, 3, and 5 years.

**FIGURE 2:**
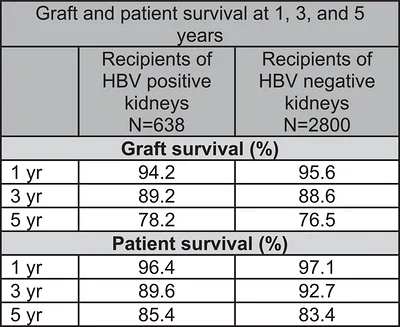
Graft and Patient survivals at 1, 3, and 5‐years post‐transplant.

**FIGURE 3:**
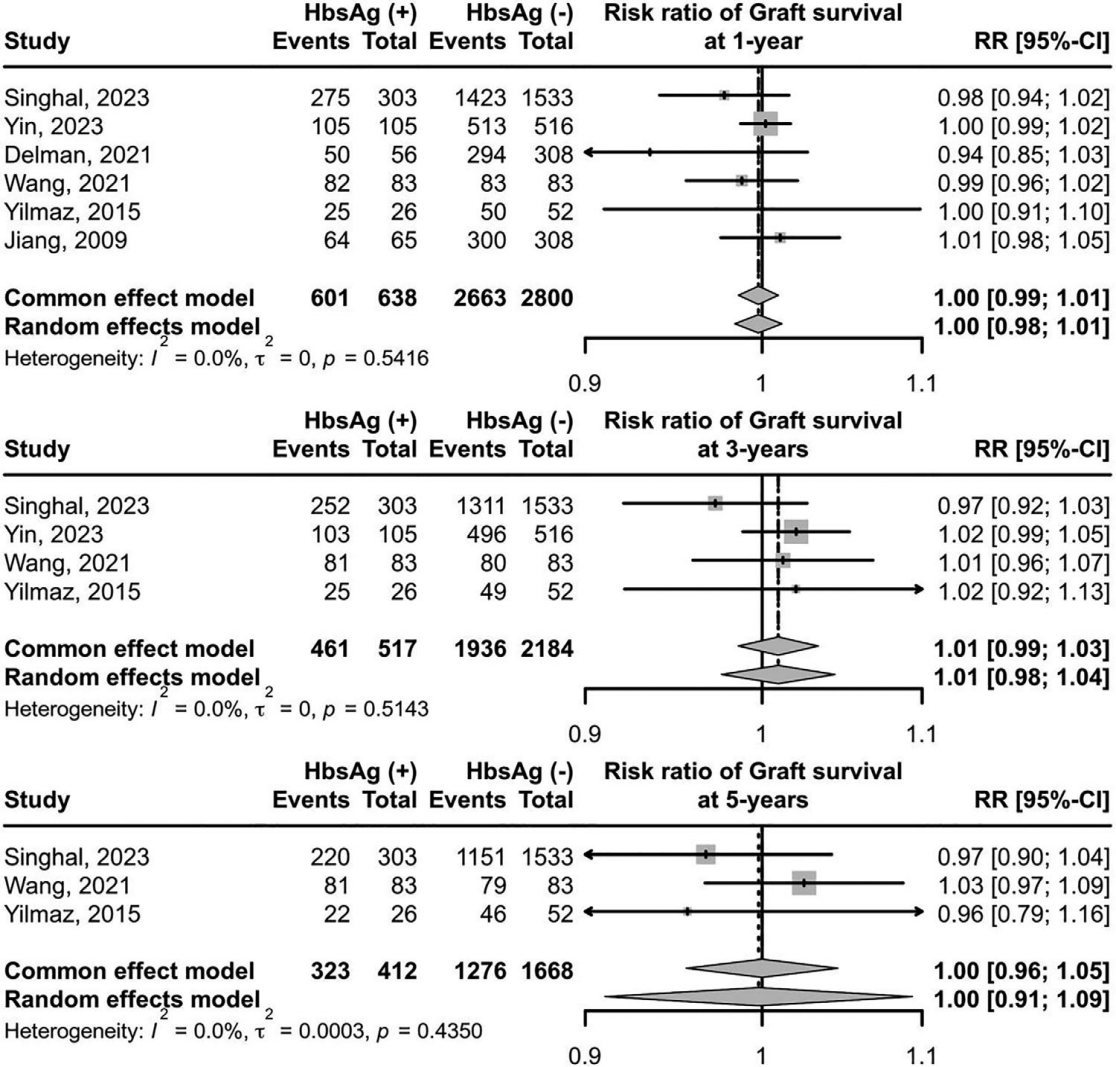
Risk Ratio of Graft Survival.

In the subgroup analysis separating the US from the Chinese studies, the relative risks of allograft survival were not statistically different when looking at the US studies alone (0.97 [0.8–1.18], 0.97 [0.92–1.03], and 0.97 [0.9–1.04] at 1‐, 3‐, and 5‐years post‐transplant) and at the Chinese studies alone (1 [0.92–1.02], 1.02 [0.97–1.06], and 1.03 [0.97–1.09] at 1‐, 3‐, and 5‐years post‐transplant) (Figure S1).

#### 3.2.2 Patient Survival Rates

The RRs of patient survival in the HBsAg or NAT positive group were not statistically different compared to those of the control group. The patient survival rates at 1‐, 3‐, and 5‐years post‐transplant were 96.4%, 89.6%, and 85.4% in recipients of HBsAg or NAT positive kidneys and 97.1%, 92.7% and 83.4% in the control group (Figure 2). The RR of patient survival in the HBsAg or NAT positive group compared to the control group was 1 [0.98–1.01], 1 [0.99–1.01] and 0.98 [0.90–1.06] at 1‐, 3‐, and 5‐years post‐transplant, respectively (Figure 4). The patient survival showed low heterogeneity, I^2^ of 0% at 1 and 3 years (0%), and of 6.8% 5 years. The subgroup analysis based on the geographical location of the study revealed that the RR of patient survival at 1,3, and 5 years remained not statistically different when looking at the Chinese studies alone (1 [0.96–1.04], 1 [0.93–1.08] and 0.96 [0.91–1.02] at 1‐, 3‐, and 5‐years post‐transplant) and the US studies separately (0.98 [0.93–1.04], 1 [0.96–1.04] and 1 [0.95–1.06] at 1‐, 3‐, and 5‐years post‐transplant (Figure S2).

**FIGURE 4:**
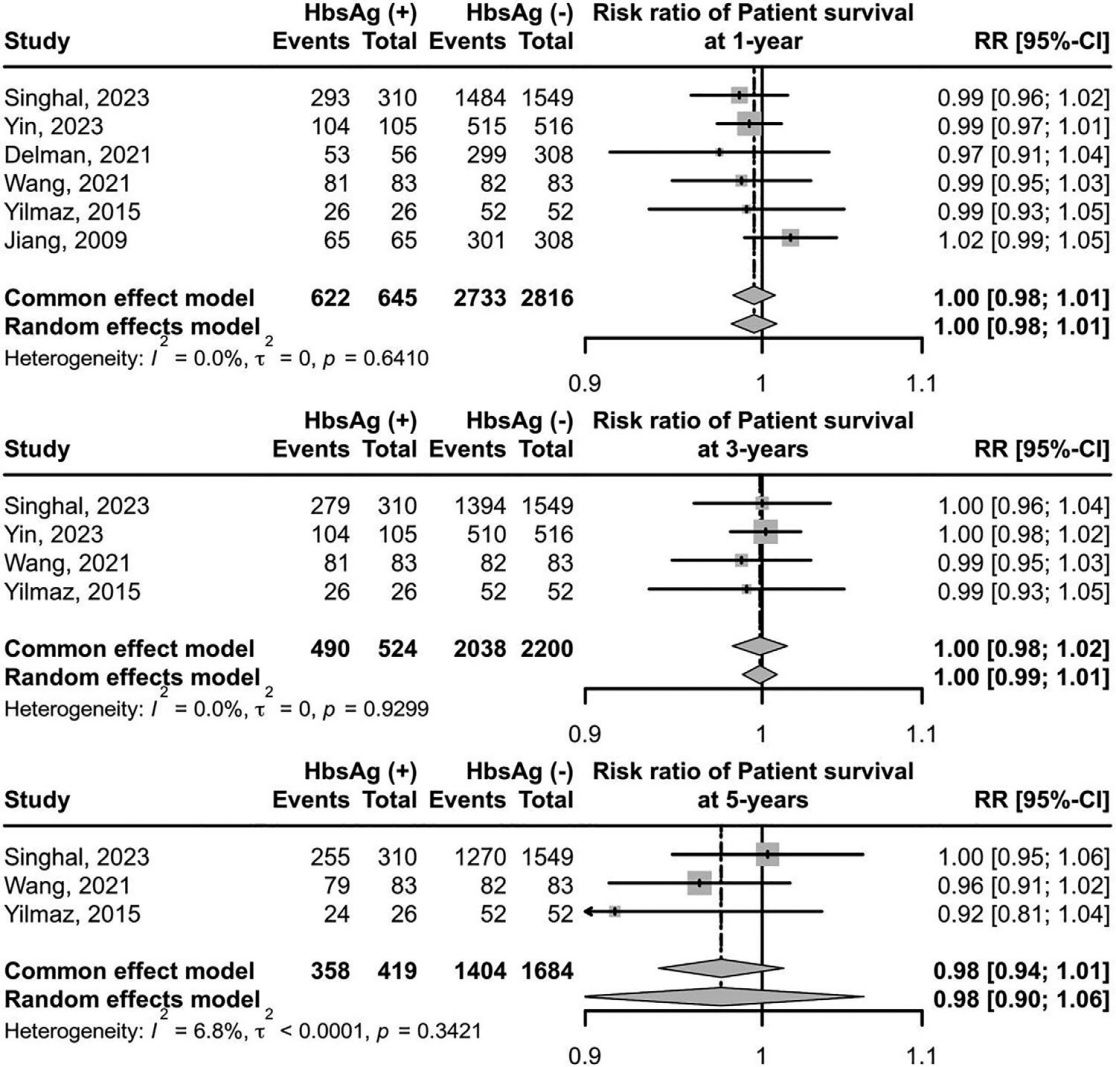
Risk Ratio of Patient Survival.

## 4 Discussion

The use of HBV positive kidneys is an opportunity to increase the donor pool. In the US, very few kidney transplants from donors with HBsAg positivity or hepatitis B viremia are being performed and the discard rate of such kidneys is high. According to the US OPTN 2017 Annual Report, the proportion of donor HBsAg positive to recipient HBsAg negative was 0.0% in DD KTRs and 0.5% in living donor KTRs [19]. A recent retrospective cohort study based on OPTN data from 2000 to 2019 reported a discard rate of 40% for HBsAg positive kidneys in 2019, which was 15% higher than the 25% discard rate of HBsAg negative kidneys [20]. Similarly, a recent retrospective UNOS database cohort study which examined outcomes of HBV positive kidney allografts between 1999 and 2021 revealed that only 0.24% of retrieved kidneys were HBV positive and of these, only 35% were transplanted [6]. The low number of retrieved kidneys and the high discard rate potentially represent missed opportunities for expanding the DD kidney pool, given that there are currently 850,000 people in the US living with chronic HBV and that the prevalence of HBsAg positivity can be as high as to 10% in certain Asian countries [21]. In addition, the use of HBV kidneys may allow certain candidates to be transplanted earlier, cutting time on dialysis shorter. In a retrospective UNOS database cohort study by Singhal and colleagues, recipients of HBV positive (HBsAg or NAT positive) kidneys had a median waitlist time of 375 days versus 625 days for recipients of HBV negative kidneys. These HBV‐positive kidneys came from regional or national sharing and had longer cold ischemia times, suggesting that local centers were more likely to decline these offers [6]. Similarly, in the study by Delman and colleagues, recipients of HBV NAT+ kidneys had significantly shorter times on the waiting list with a median of 283.4 versus 424.2 days (*p* < 0.01) [5]. In addition, recipients of HBV NAT+ kidneys spent an average of 1.9 years less on dialysis than recipients of HBV negative kidneys [5].

### 4.1 Allograft and Patient Survivals

Available literature suggests similar graft and patient survival among patients who received HBV positive kidneys compared to recipients of HBV negative kidneys. In the same retrospective cohort study mentioned above [6], 352 KTRs of HBV positive kidneys had 10‐year patient and graft survival rates that were similar to those who received HBV negative kidneys. Interestingly, a subgroup analysis of 58 HBsAb negative KTRs of HBV positive kidneys revealed that they did as well as those who received HBV negative kidneys. The authors thus advocated for the use of HBV positive kidneys even in recipients who were not immune, as long as appropriate prophylaxis and antiviral therapies were administered post‐transplant. However, this paper did not include information on the rate of viral transmission, HBsAg conversion, or development of clinical hepatitis, and thus provides limited data related to the safety of this practice. Other smaller studies have also led to similar conclusions. Delman and colleagues reported their prospective experience with 57 KTRs who were HBV seronegative at the time of transplant and received a HBV NAT positive kidney between 2017 and 2020 followed by entecavir therapy for one year post transplant [5]. Their one‐year patient and allograft survival were similar to that of recipients of HBV NAT negative kidneys during the same time period. Similarly, a large Chinese retrospective cohort study comprising 105 HBsAg negative recipients who received kidneys from HBsAg+ donors between 2017 and 2020 reported similar graft and patient survival rates when compared to recipients of HBcAb+ kidneys [7]. The current meta‐analysis reveals similar patient and allograft survival at 1, 3, and 5 years in 638 recipients of HBsAg or NAT+ kidneys compared to 2800 recipients of HBsAg and NAT negative kidneys. This finding suggests that the quality of HBV positive kidneys is not inferior to the standard of care HBsAg and NAT negative kidneys, and that the potential risks of transmission, hepatitis, or hepatocellular carcinoma do not seem to impact mortality risk in the short or mid‐term. HBV genotype prevalence differs in the US and in China with subsequent differences in natural history of the infection which might differentially affect the post transplant outcomes. Genotype C is more prevalent in Asia and is more likely to be associated with cirrhosis and HCC, as opposed to genotype A which is more prevalent in the US [22]. When we performed subgroup analyses of allograft and patient survival based on the geographical location of the study, there did not seem to be a lower RR of allograft and patient survival in recipients of HBV positive kidneys in China, indicating that the HBV positive kidneys were not associated with a lower patient survival, even in China where the more oncogenic genotype C is more prevalent. However, survival alone is not sufficient to establish safety, particularly with limited follow‐up time. Long‐term hepatic outcomes, chronic infection, and hepatocellular malignancy risk were largely unreported in the included studies and while the short and mid‐term survivals seem comparable, we currently lack long term patient and allograft survival data related to this practice.

### 4.2 Risk of Viral Transmission and Clinical Hepatitis

The concern for viral transmission may be a barrier to the use of the HBV positive kidneys. Few studies have commented on the safety of this practice and on the strategies that could mitigate the potential risks of hepatitis B viremia and denovo hepatitis. Our review found that the risk of viral transmission was low (4.9%), especially in recipients who are immune against HBV with pre‐transplant HBsAb titers of >100 mIU/mL and that the use of prophylaxis (HBIG and/or antivirals) can further reduce this transmission risk, even in nonimmune recipients. We hereby summarize some key studies by chronological order, keeping in mind that earlier studies included immune recipients and donors that were HBsAg positive but not viremic, and that subsequent more contemporary ones had more liberal criteria and included non‐immune recipients as well as viremic NAT positive donors.

In a single center prospective nonrandomized controlled Chinese study from 2009, 65 immune (HBsAb positive) candidates received a HBsAg positive kidney, with HBIG given on the day of transplant and at 1‐month post‐transplant [14]. Over a mean follow‐up period of 30 months, only 1 recipient (1.5%) with pre‐transplant HBsAb titer of 63.6 IU/L developed denovo HBsAg without HBV viremia or liver injury. Treatment with lamivudine led to HBsAg loss. In addition, 7 anti‐HBc negative recipients pre‐transplant developed anti‐HBc antibodies, implying a possible immunological response to HBV exposure. Seven recipients of HBV NAT+ kidneys received weekly HBIG and lamivudine for 3 months. These recipients were all anti‐HBsAb+ and anti‐HBc+, and they did not develop HBsAg positivity nor HBV viremia.

In 2014, a Thai study reported a retrospective longitudinal experience of 43 HBV‐immune (HBsAb titers of >100mIU/mL) recipients of DD HBsAg+ but NAT‐ kidneys transplanted between 2000 and 2013 [16]. Fifty‐four percent of the recipients received prophylaxis (lamivudine ± HBIG) at the time of transplant. In this study, none of the 43 recipients developed HBsAg positivity, HBV viremia, or anti‐HBc seroconversion. With the combination of immune recipients and non‐viremic HBsAg+ kidneys, the rate of transmission was assessed to be negligible, even in the absence of peri‐transplant prophylaxis.

More contemporary studies included donors who were viremic and recipients who were not immune. In the Delman study from 2021, all the recipients of NAT+ kidneys received entecavir prophylaxis for a year [5]. Sixteen percent of the recipients of NAT+ kidneys developed transient HBV viremia without clinical hepatitis, which resolved after a median of 80 days of entecavir, and none of the patients were viremic at one year. This study is of particular relevance because it included donors who were viremic in addition to being HBsAg+, as opposed to the majority of the earlier studies. In addition, 32% of the recipients of NAT+ kidneys included in this study were HBsAb‐ despite vaccination, and were thus deemed to be susceptible to infection. This paper did not compare the incidence of HBV viremia between immune and non‐immune recipients. However, the fact that none of the recipients were viremic at one year despite donor viremia and despite the absence of protective immunity at the time of transplant is very encouraging. Similarly, Lopez‐Soler et al. from the US reported their experience in transplanting 16 NAT+ kidneys from DDs into immune elderly recipients with entecavir prophylaxis for 52 weeks post transplant. While 3 patients developed transient viremia at a median of 3 days post transplant with a median viral load of 83 IU/mL, none of the patients were viremic at 9 and 12 months post transplant [9]. It is important to note though, that both studies only provided one‐year follow up data while recipients were still on entecavir. In a subsequent follow up paper from the Delman group which included 123 recipients of NAT positive kidneys with a longer follow up duration, 4.6% of the recipients of NAT positive kidneys developed HBV viremia at a median follow up of 24 months post transplant with equivalent time to detection of 28 months, but 100% of the patients had an undetectable HBV viral load at the time of last follow up [23]. This data highlights that the optimal duration of prophylaxis is yet to be determined, that late transmission after discontinuation of the anti‐viral prophylaxis could still happen, and that close monitoring of HBV viremia and HBsAg status might be warranted for several years post‐transplant in recipients of HBV NAT positive kidneys. It is also worth noting that those 2 US studies of viremic NAT+ donors did not include HBIG as part of their prophylactic strategies, probably in light of the emerging evidence suggesting a limited role of HBIG when coupled with antivirals in liver transplant recipients [24, 25, 26]. Nevertheless, none of the patients were viremic at the time of last follow up despite the lack of HBIG prophylaxis. In contrast, another Chinese study reported the outcomes of 83 HBsAg‐ recipients who received HBsAg+ living donor kidneys [8]. Twenty out of sixty three recipients (25%) were not immune (HBsAb‐) at the time of the transplant. Despite receiving prophylaxis with either HBIG or antivirals or both, 2 non‐immune recipients developed HBsAg positivity with subsequent transaminitis and one of them died of liver failure 8 months post transplant. One of these patients received an HBsAg+ but NAT‐ kidney, which highlights the importance of recipient immunity in preventing viral transmission, even in the absence of donor viremia. It is important to note that the first recipient of a non‐viremic kidney only received lamivudine prophylaxis for 1.5 months and the second recipient of a viremic kidney only received one dose of HBIG 2,000 units as well as lamivudine for 2 months, which begs the question if the course could have been altered by a longer antiviral course that would have been continued until the recipient could have been vaccinated and developed subsequent immunity.

A US single center experience of using HBV viremic donors in 8 KTRs between 2017 and 2020 examined anti‐HBc seroconversion at one year [15]. All recipients of viremic kidneys were immune with HBsAb titers >10 mIU/mL and 62% of them (5 patients) received prophylactic entecavir or lamivudine at the time of the transplant. While 62% of the recipients had anti‐HBcAb seroconversion at an average of 47 days post‐transplant, none of them developed HBsAg positivity throughout the study. Two patients had a positive viral load under the threshold of detection on one occasion, and it cleared on subsequent testing. The 2 patients who had anti‐HBc seroconversion and were not on prophylactic antivirals at the time of transplant were started on entecavir upon seroconversion. The other 2 patients who had anti‐HBc seroconversion despite prophylaxis had HBsAb titers of <100 mIU/mL at the time of transplant. In light of these results, and in light of the emerging literature suggesting that pre‐transplant titers of >100 mIU/mL might confer more protection against potential infction, the authors have since changed their institutional policy to only include candidates with HBsAb titers of >100 mIU/mL pre‐transplant and to include systematic prophylactic antiviral therapy for all recipients of HBV viremic kidneys. It is important to note though that anti‐HBc seroconversion might just be a reflection of exposure and might imply an immunological response to HBV exposure, rather than a clinically significant infection. However, this scenario still warrants close post transplant monitoring, similar to the monitoring of recipients who are HBcAb+ at the time of the transplant, who are known to have a reactivation rate of 2%–12% [27, 28].

In the largest Chinese retrospective cohort study to date, all 105 recipients of HBsAg+ kidneys received antiviral prophylaxis with HBIG and/or antiviral drugs [7]. In this study, 79% of the donors were viremic. The de novo hepatitis rate was 3.8% (4 patients) versus 0.4% in the control group. The 4 patients who developed de novo hepatitis achieved HBsAg clearance after treatment. More than 50% of the patients received living donor kidneys and the living donors were treated with anti virals prior to donation for a median duration of 7 months. This resulted in a much lower donor HBV viral load at the time of transplant although not complete viral suppression. Seventy‐five percent of the recipients were HBsAb+ prior to transplant and the rest were not immune. All the recipients received post‐transplant antiviral prophylaxis. It is important to note that 3 out of the 4 patients who developed de novo hepatitis were HBsAb‐ prior to transplant, again highlighting the fact that the risk of transmission or denovo hepatitis seems to be low in immune recipients who receive prophylaxis, even in the presence of donor viremia. More importantly, despite the potential transmission risk, all the infected recipients had HBsAg loss after treatment, which is comparable to the natural history of HBV in the general immunocompetent population. The authors have since developed a stratified antiviral prophylactic strategy based on recipient's HBsAb titers and HBV NAT status of the donor which is being studied in a multicenter prospective cohort study (clinical trials.gov NCT04562051). The details of their strategy are beyond the scope of this paper, but it is worth mentioning that their strategy does not include HBIG nor antivirals in HBV immune recipients with titers >100 mIU/mL who are receiving HBsAg+ NAT‐ kidneys.

### 4.3 Recommendations and Future Directions

Based on this systematic review of the literature, we conclude that the risk of early HBV transmission seems to be low in immune recipients with HBsAb titers of >100 mIU/mL coupled with antiviral prophylaxis ± HBIG, with close serial HBV DNA, HBsAg, and HBsAb monitoring, at select interval post‐transplant. The very low rate of transmission of less < 5% included immune and non immune recipients and is expected to be much lower when considering immune HBsAb+ recipients only. Despite the very low rate of transmission of < 5% in immune and non immune recipients, the vast majority of the recipients who developed early viremia post transplant were no longer viremic at the time of last follow up, which is very reassuring. The only reported case of liver failure leading to death occurred in a recipient who was not immune at the time of transplant and who only received post transplant prophylaxis with HBIG 2000 units x 1 dose and lamivudine for 2 months post transplant, an approach that would no longer be recommended nowadays. Most centers using HBV positive kidneys have been using nucleotide analogs for a minimum of one year post transplant. The one year duration of antiviral prophylaxis is based on studies utilizing isolated HBcAb seropositive donors for non‐hepatic transplants, yet its generalizability to HBV NAT‐positive donors is unclear.

Optimizing the recipients can theoretically be achieved by ensuring that all waitlisted patients get vaccinated pre‐transplant, which is in line with the Center for Disease Control and Prevention recommendations that support HBV vaccination for all adults aged 19–59 and for those ≥60 with risk factors including patients on chronic dialysis [29]. Unfortunately, the literature suggests that more than 40% of dialysis patients receiving the standard dose of the HBV vaccine are non‐responders due to impaired immune response, and the World Health Organization has since recommended several ways to improve the immune response rate in patients on dialysis including: (1) giving more than 3 doses of the standard vaccine, (2) giving a higher dose or double dose of the standard vaccine with each injection, or (3) giving the newer recombinant more potent adjuvented vaccine (Heplisav‐B) [30]. The latter strategy has the added benefit of a shorter immunization schedule, as it only entails 2 doses of the vaccine given one month apart.

In light of the above results, our centers have implemented a protocol to guide the use of the HBV positive organs (HBsAg or NAT+) for kidney transplant candidates. We are starting with a conservative approach and only considering immune candidates with documented HBsAb titers of >10 mIU/mL while on the waitlist. We administer intra‐operative HBIG 10,000 units at the time of the transplant and start entecavir for all recipients of HBV positive kidneys, until the admission pre‐operative HBsAb titer results, usually within 24–48 h. Entecavir is discontinued if the pre‐transplant HBsAb titer is >100 mIU/mL. The HBsAb titers are followed serially post‐transplant, with plans to resume entecavir and vaccinate with Heplisav‐B at 4 months post‐transplant if the titers fall under 40 mIU/mL. If the pre‐transplant HBsAb titers are <100 mIU/mL, entecavir is continued for at least 6 months, until the recipient can be vaccinated at 4 months post‐transplant and mount a post‐vaccination HBsAb titer of more than 100 mIU/mL. If the HBsAb titer remain <100 mIU/ml despite vaccination, entecavir is continued for 1 year post transplant. We monitor HBV DNA, HBsAg, and HBsAb titers at 1, 3, 6, 9, and 12 months post‐transplant, and every 6 months thereafter for the first 3 post‐transplant years. Since this approach is not considered part of our standard transplant care, eligible immune recipients are asked to sign a separate consent that goes over the low theoretical risk of transmission that is mitigated by pre transplant immunity and post‐transplant prophylaxis.

### 4.4 Limitations

Our review and meta‐analysis has several limitations including the lack of homogeneity amongst the different studies included. Most importantly, majority of the studies included in the meta‐analysis did not separate recipients of HBsAg+NAT‐ donors from those of NAT+ donors and those 2 clinically different scenarios might not carry the same transmission risk and might subsequently be associated with different patient and allograft survivals. Besides the limited Delman, Lopez‐Soler, and Carnemolla US experiences (56, 16, and 8 patients), the Yin Chinese retrospective experience, and a smaller study from Turkey of 26 recipients of viremic donors, the vast majority of the recipients included in the meta‐analysis received HBsAg+NAT‐ kidneys. In addition, not all qualitative NAT+ donors confer the same transmission risk. A recent retrospective single study from the US including 25 non hepatic solid organ recipients of qualitative NAT+ donors revealed that 36% of the NAT+ donors had undetectable viral loads and the risk of transmission from such donors was felt to be negligible [31]. The authors thus recommended quantifying the viral load for all HBV NAT+ organs at the time of the organ offers, in an attempt to risk stratify the recipient's risk of donor derived HBV infection, which might then help determine the appropriate post prophylactic strategies. We were unable to perform subgroup analyses pertinent do the viremic status of the donor (HBsAg+NAT+ versus HBsAg+NAT‐) due to inconsistencies in reporting between the different studies, and the pooled survival estimates could have masked meaningful differences in risk across these subgroups. In addition, the prophylactic strategies were very variable among different studies and were not standardized within a given study. The use of HBIG was very inconsistent and there were no head‐to‐head comparison of protocols including HBIG + antivirals versus antivirals alone, and we are thus unable to comment on the utility of HBIG coupled with antivirals. We do note though that the use of HBIG in liver transplantation has recently decreased as it was felt to have limited efficacy when coupled with potent nucleotide analogues [24, 25, 26]. The studies commenting on the transmission risk mostly provided short term follow up while recipients were still on oral antivirals and we are thus unable to comment on the optimal duration of prophylaxis post transplant and on the risk of transmission following discontinuation of the nucleotide analogs. As a result, and due to the incertitude related to long term transmission risk, some centers are deliberately limiting this practice to immune elderly recipients with limited qualifying wait time, who can then balance the risk of not being transplanted with the potential and theoretical low risk of transmission [9]. Finally, there could have been a selection bias inherent to observational studies, that could have contributed to the comparable survival between the 2 groups. HBV positive kidneys could have been of better quality which could have accounted for the similar allograft outcomes among recipients of HBV positive kidneys and those of HBV negative kidneys, but most of the included studies did not comment on the quality of the kidneys (Donation after cardiac death versus Donation after brain death, KDPI, donor age, creatinine at the time of death…) between patients that received standard of care kidneys and those who received HBV positive kidneys. Similarly, there could have been a selection bias on the recipient's end that could have conferred a survival benefit and accounted for the similar patient and allograft survivals in recipients of HBV positive and those of HBV negative kidneys. Individual centers could have had certain exclusion criteria that precluded elderly sicker recipients to receive HBV positive kidneys, but this level of granularity was not included in the papers reviewed.

We acknowledge that the patient and allograft survival outcomes or the transmission risk could have been different between donors that were HBsAg+NAT‐ and those who were NAT+, and between recipients that were immune going into the transplant and those who were HBsAb‐, and that the different non standardized prophylactic strategies adopted by every individual transplant center, could have affected the transmission risk. For this reason, and to mitigate the transmission risk which seems to be low in immune recipients, our center is deliberately limiting this practice to recipients that are HBsAb+ going into the transplant, and we are deliberately resuming the prophylaxis in immune recipients whose titers wane over time because of post‐transplant immunosuppression. Finally, there remain several questions that we did not address in this review, including the optimal duration of post‐transplant prophylaxis and monitoring, and whether this practice can be safely generalized to non‐immune HBsAb‐ transplant candidates or to select population including candidates that are co infected with HCV or HIV. We also could not comment on the long‐term risk of cirrhosis and HCC due to limited follow up data, but we assumed this risk to be low in the absence of detectable viremia at the time of last follow up.

## 5 Conclusion

In conclusion, the use of HBV positive (HBsAg+ or NAT+) kidneys appears to be a reasonably safe practice with a low short term risk of HBV transmission (4.9%) or HBV‐related complications, especially in HBV immune recipients and when using prophylactic antivirals ± HBIG. The use of such kidneys might prove to be an effective and viable strategy to help address the organ shortage and to reduce waiting times prior to transplant, without compromising allograft and patient survival, especially in select recipients who do not have enough qualifying wait time and who have preexisting immunity against hepatitis B (HBsAb+). The most optimal and cost‐effective antiviral prophylactic strategies are yet to be determined as this practice gains popularity amongst transplant centers. Ultimately, informed consent of the recipients and shared decision making are necessary and will need to balance the non negligeable risk of mortality on dialysis against the potential low theoretical risk of transmission which seems to be further mitigated by pre transplant immunity, post‐transplant antiviral prophylaxis, as well as close monitoring of HBV serologies at select post‐transplant interval, especially after discontinuation of anti‐viral agents. Vaccinating all wait listed patients prior to transplant should be considered to minimize the transmission risk. Quantifying the donor viral load at the time of organ offer might help stratify the risk of donor derived infection and guide the optimal post transplant prophylactic strategy and duration.

## Ethics Statement

Ethics committee approval was not required for this study, as it is a systematic review and meta‐analysis of previously published data and did not involve direct participation of human subjects or collection of identifiable patient information. Informed consent was therefore not applicable.

## Conflicts of Interest

The authors declare no conflicts of interest.

## Supporting information




**Supporting Information**: ctr70733‐sup‐0001‐FigureS1.jpeg

**Supporting Information**: ctr70733‐sup‐0002‐FigureS2.jpeg

## Data Availability

The data that support the findings of this study are available from the corresponding author upon request.
